# Draft genomes of fluoroquinolone-resistant *Salmonella enterica* subspecies *enterica* strains isolated from Cambodian poultry marketplaces

**DOI:** 10.1128/mra.00591-24

**Published:** 2025-01-16

**Authors:** Evelyn C. Goh, Dalong Hu, Rortana Chea, Adrian Low, Xiu Qi Koh, Sothyra Tum, Yann Felix Boucher

**Affiliations:** 1 Saw Swee Hock School of Public Health, National University of Singapore and National University Health System, Singapore, Singapore; 2 Singapore Centre for Environmental Life Sciences Engineering (SCELSE), National University of Singapore, Singapore, Singapore; 3 National Animal Health and Production Research Institute (NAHPRI), General Directorate of Animal Health and Production, Phnom Penh, Cambodia; 4 Department of Medicine, Yong Loo Lin School of Medicine, National University of Singapore, Singapore, Singapore; University of Maryland School of Medicine, Baltimore, Maryland, USA

**Keywords:** genome, fluoroquinolone, resistant, antimicrobial, Cambodia, *Salmonella enterica*, poultry, animal-to-human transmission, marketplace

## Abstract

High-quality draft genomes of six *Salmonella enterica* subspecies *enterica* strains from Cambodian poultry marketplaces were sequenced. The strains were identified as Corvallis-, Monschaui-, and Kentucky-serovars. The fluoroquinolone resistance gene, *qnrS,* was found in three strains in different Cambodian provinces. These genomes serve as references for antibiotic resistance genotypes.

## ANNOUNCEMENT

**Fig 1:**
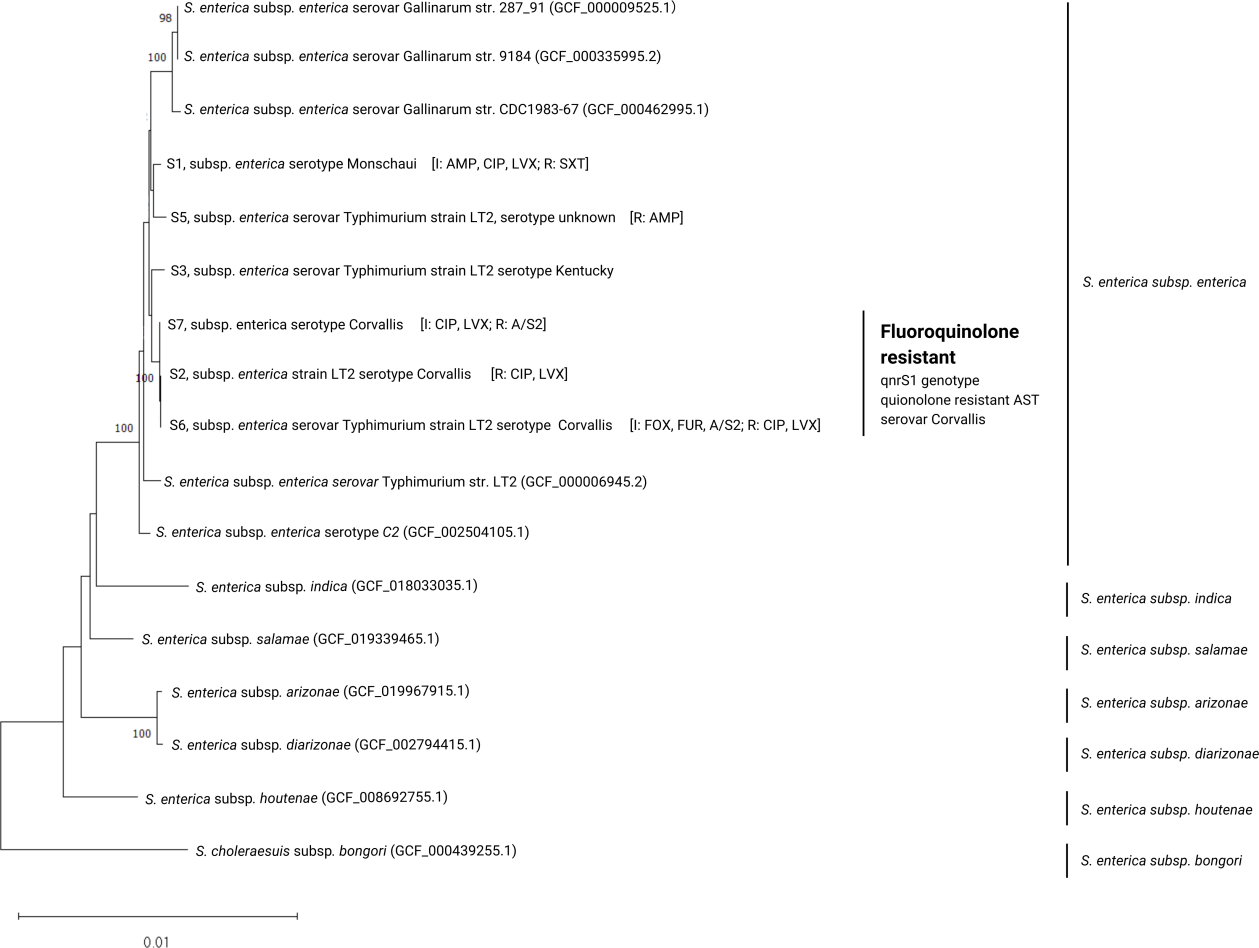
A maximum likelihood tree of *Salmonella* species and the six major *Salmonella enterica* subspecies *enterica* (21) based on core-genes (2,052). Numbers beside branch nodes indicate bootstrap percentage after 1,000 replications in constructing the tree. Bootstrap of 1,000 replicates were calculated, and values lower than 70% are not shown. The phylogenetic tree is rooted to the *Salmonella choleraesuis* subspecies *bongori* (22). Scale bar corresponds to 0.01 nucleotide substitutions per site.

Animal-to-human transmission of antimicrobial-resistant (AMR) bacteria can occur in poorly regulated livestock market chains in Cambodia and exacerbated by antimicrobial misuse (1, 2). Animal ethics has been exempted by the Ministry of Health Cambodia as work was performed by authority, General Directorate of Animal Health and Production, Cambodia. Fifty retailers in three provinces (Phnom Penh; Speu; Takeo) were recruited and interviewed to understand their antimicrobial usage, and information was stored on RedCap (3). Physical samples of pooled cloacal swabs from five healthy chickens and pooled skin swab samples from five chicken carcasses were collected. *Salmonella* strains were isolated as previously described (4). Six *Salmonella* strains were tested for antimicrobial susceptibility testing (AST) via broth microdilution method (5) and interpreted according to standard guidelines (6). Cultures were processed in Singapore for genomic sequencing. Cultures of 5 mL Lysogeny broth (LB; 10 g/L tryptone, 5 g/L yeast extract, and 10 g/L NaCl) were incubated at 37°C shaking at 150 rpm for 12 h. The PowerFecal-Pro kit (Qiagen) was used for DNA extraction as per manufacturer’s protocol. DNA Library was constructed using an Illumina TruSeq Nano DNA Library Prep kit for 150 bp paired-end sequencing on an Illumina HiSeqX sequencer. Default parameters for all data processing were adopted unless stated otherwise. Trimmomatic-v0.39 (7) was used to remove adaptors and low-quality reads. The Shovill pipeline https://github.com/tseemann/shovill that wraps SPAdes-v3.15.5 (8) and Pilon-v1.2.4 (9) was used for assembly and polishing, respectively. Annotation of CDS and rRNA genes of the draft genomes were done using Prokka-v1.14.5 and Barrnap-v0.9 (10). SeqSero2-v1.1.0 was used to identify serotypes (10). 16S rRNA genes were searched against the NCBI rRNA/ITS database (excluding uncultured sequences) to identify close relatives (11). Core-genes among representative genomes of each *S. enterica* subspecies and closely related serotypes were identified using Roary-v3.11.2 (11), and the concatenated MUSCLE-aligned core-genes were used to build a maximum-likelihood tree using MEGA-v11 (12). Average nucleotide identities based on BLAST+ (ANIb) were calculated using JSpeciesWS-v1.2.1 (13). Staramr (14) and the Resistance Gene Identifier-v6.0.3 (15) were used to identify antimicrobial resistance profiles. CheckM-v1.2.2 was used to assess genome quality (16). Table 1 summarizes the genomic statistics and characteristics of the six genomes. Draft genomes (non-circularized) were assembled from short-read sequences with a minimum coverage of 210×. The mean genome size is 1.30 ± 0.14 (SD) Mbp of 100% completeness, ≤0.5% contamination, and the majority of genomes can be considered high quality (17). Strains S2, S6, and S7 possess *qnrS1,* conferring resistance to fluoroquinolone (18). All strains clustered in the same clade as *Salmonella enterica* subsp. *enterica* strain LT2 (Fig. 1) (19). Phylogenetic proximity among these three strains suggests a close genetic relationship, corroborated by AST which confirms their resistance to ciprofloxacin and levofloxacin. This indicates the potential spread of AMR *S. enterica* between Cambodian provinces from poultry.

**TABLE 1:** Sequencing and statistics of six draft genomes

Taxon	*Salmonella enterica* subsp. *enterica*	*Salmonella enterica* subsp. *enterica* strain LT2	*Salmonella enterica* subsp. *enterica* serovar Typhimurium strain LT2	*Salmonella enterica* subsp. *enterica* serovar Typhimurium strain LT2	*Salmonella enterica* subsp. *enterica* serovar Typhimurium strain LT2	*Salmonella enterica* subsp. *enterica*
*Predicted serotype*						
(*Kauffman-White scheme,* *SeqSero 2.0*)	Monschaui	Corvallis	Kentucky	–^*a*^	Corvallis	Corvallis
*Predicted MLST*						
(*Achtman, Staramr*)	4,025	1,541	314	423	1,541	1,541
*16S rRNA gene BLAST %*	–	100% (835 bp)	98.97% (978 bp)	99.74% (1,528 bp)	100% (642 bp)	–
*Identity (sequence size*)						
*Province/source*	Phnom Penh/shop + live chicken cage	Phnom Penh/chicken meat	Phnom Penh/chicken cloaca	Phnom Penh/chicken cloaca	Takoe/chicken meat	Takoe/chicken meat
*AMR profile (Staramr*)	Sensitive	Ciprofloxacin I/R	Sensitive	Sensitive	ciprofloxacin I/R	Ciprofloxacin I/R
*AMR genotype (Staramr*)	None	qnrS1	None	None	qnrS1	qnrS1
*AST results*	I: ampicillin, ciprofloxacin, levofloxacin R: trim/ethoprim/sulfameth oxazole (SXT)	R: ciprofloxacin, levofloxacin	–	R: ampicillin	I: cefoxitin (FOX), cefuroxime (FUR), ampicillin/sulbactam 2:I ratio (A/S2) R: ciprofloxacin, levofloxacin	I: ciprofloxacin, levofloxacin R: ampicillin/sulbactam 2:1 ratio (A/S2)
*Genome size (bp*)	4,845,063	4,984,147	4,955,474	4,822,243	4,983,031	5,179,146
*Total raw paired-end reads*	4,981,093	3,751,393	5,069,163	4,434,725	4,616,947	4,999,161
*Coverage × (filtered read count ×*						
*read length) / total genome size*	291	210	287	256	259	269
*No. of CDS*	4,516	4,727	4,658	4,555	4,733	4,969
*Protein coding, %*	98.39	98.27	98.25	98.36	98.32	98.36
*rRNAs (5S, 16S, 23S*)	(4, 1, 0)	(6, 1, 0)	(4, 2, 0)	(1, 1, 5)	(6, 1, 0)	(8, 0, 0)
*tRNAs*	68	75	76	68	73	74
*G + C content (%*)	52.1	51.9	52	52.1	51.9	51.7
*No. of contigs*	38	48	34	48	51	39
*N50 assembled genome*						
(*bp*)	327,738	523,595	518,578	178,153	402,915	520,963
*CheckM completeness*						
(*%*)	100.00	100.00	100.00	100.00	100.00	100.00
*CheckM contamination*						
(*%*)	0.33	0.14	0.15	0.33	0.14	0.50
*Biosample/ SRA/assembly accession numbers*	SAMN38755249/SRS19884810/GCA_038433325.1	SAMN38755250/SRS19884811/GCA_038433315.1	SAMN38755251/SRS19884812/GCA_038433385.c1	SAMN38755252/ SRS19884813/ GCA_038433405.1	SAMN38755253/ SRS19884814/ GCA_038433415.1	SAMN38755254/ SRS19884815/ GCA_038433425.1

^*a*^ “–”, means no resistance detected after antibiotic susceptibility testing.

## Data Availability

Raw sequences and assembled genomes were deposited to the Sequence Read Archive under BioProject number PRJNA1043807. Individual BioSample accession numbers have been listed in Table 1.

## References

[1] Chea R. 2023. Foodborne bacteria in the Cambodian meat value chain: emphasis on the risk of Salmonella in chicken and pork from traditional markets to household consumption. Acta Uni Agric Suec 8. doi:10.54612/a.u711g61ocj

[2] Schwan CL, Desiree K, Bello NM, Bastos L, Hok L, Phebus RK, Gragg S, Kastner J, Vipham JL. 2021. Prevalence of Salmonella enterica isolated from food contact and nonfood contact surfaces in Cambodian informal markets. J Food Prot 84:73–79. doi:10.4315/JFP-20-112 33393619

[3] Harris PA, Taylor R, Minor BL, Elliott V, Fernandez M, O’Neal L, McLeod L, Delacqua G, Delacqua F, Kirby J, Duda SN. 2019. The REDCap consortium: building an international community of software platform partners. J Biomed Inform 95:103208. doi:10.1016/j.jbi.2019.103208 31078660 PMC7254481

[4] Organization IS. 2017. Microbiology of the food chain‐horizontal method for the detection, enumeration and serotyping of Salmonella‐Part 1: detection of Salmonella spp. Geneva, Switzerland International Organization for Standardization Geneva

[5] CLSI. 2018. Performance standards for antimicrobial disk and dilution susceptibility tests for bacteria isolated from animals. Clinical and Laboratory Standards Institute VET01, Wayne, PA.

[6] CLSI. 2018. Performance standards for antimicrobial disk and dilution susceptibility tests for bacteria isolated from animals. Clinical and Laboratory Standards Institute VET08, Wayne, PA.

[7] Bolger AM, Lohse M, Usadel B. 2014. Trimmomatic: a flexible trimmer for Illumina sequence data. Bioinformatics 30:2114–2120. doi:10.1093/bioinformatics/btu170 24695404 PMC4103590

[8] Prjibelski A, Antipov D, Meleshko D, Lapidus A, Korobeynikov A. 2020. Using SPAdes de novo assembler. Curr Protoc Bioinformatics 70:e102. doi:10.1002/cpbi.102 32559359

[9] Walker BJ, Abeel T, Shea T, Priest M, Abouelliel A, Sakthikumar S, Cuomo CA, Zeng Q, Wortman J, Young SK, Earl AM. 2014. Pilon: an integrated tool for comprehensive microbial variant detection and genome assembly improvement. PLoS ONE 9:e112963. doi:10.1371/journal.pone.0112963 25409509 PMC4237348

[10] Zhang S, den Bakker HC, Li S, Chen J, Dinsmore BA, Lane C, Lauer AC, Fields PI, Deng X. 2019. SeqSero2: rapid and improved Salmonella serotype determination using whole-genome sequencing data. Appl Environ Microbiol 85:e01746-19. doi:10.1128/AEM.01746-19 31540993 PMC6856333

[11] Morgulis A, Coulouris G, Raytselis Y, Madden TL, Agarwala R, Schäffer AA. 2008. Database indexing for production MegaBLAST searches. Bioinformatics 24:1757–1764. doi:10.1093/bioinformatics/btn322 18567917 PMC2696921

[12] Tamura K, Stecher G, Kumar S. 2021. MEGA11: molecular evolutionary genetics analysis version 11. Mol Biol Evol 38:3022–3027. doi:10.1093/molbev/msab120 33892491 PMC8233496

[13] Richter M, Rosselló-Móra R, Oliver Glöckner F, Peplies J. 2016. JSpeciesWS: a web server for prokaryotic species circumscription based on pairwise genome comparison. Bioinformatics 32:929–931. doi:10.1093/bioinformatics/btv681 26576653 PMC5939971

[14] Bharat A, Petkau A, Avery BP, Chen JC, Folster JP, Carson CA, Kearney A, Nadon C, Mabon P, Thiessen J, Alexander DC, Allen V, El Bailey S, Bekal S, German GJ, Haldane D, Hoang L, Chui L, Minion J, Zahariadis G, Domselaar GV, Reid-Smith RJ, Mulvey MR. 2022. Correlation between phenotypic and in silico detection of antimicrobial resistance in Salmonella enterica in Canada using staramr. Microorganisms 10:292. doi:10.3390/microorganisms10020292 35208747 PMC8875511

[15] Alcock BP, Huynh W, Chalil R, Smith KW, Raphenya AR, Wlodarski MA, Edalatmand A, Petkau A, Syed SA, Tsang KK, et al.. 2023. CARD 2023: expanded curation, support for machine learning, and resistome prediction at the comprehensive antibiotic resistance database. Nucleic Acids Res 51:D690–D699. doi:10.1093/nar/gkac920 36263822 PMC9825576

[16] Parks DH, Imelfort M, Skennerton CT, Hugenholtz P, Tyson GW. 2015. CheckM: assessing the quality of microbial genomes recovered from isolates, single cells, and metagenomes. Genome Res 25:1043–1055. doi:10.1101/gr.186072.114 25977477 PMC4484387

[17] Bowers RM, Kyrpides NC, Stepanauskas R, Harmon-Smith M, Doud D, Reddy TBK, Schulz F, Jarett J, Rivers AR, Eloe-Fadrosh EA, et al.. 2018. Corrigendum: minimum information about a single amplified genome (MISAG) and a metagenome-assembled genome (MIMAG) of bacteria and archaea. Nat Biotechnol 36:660. doi:10.1038/nbt0718-660a PMC7608355 29979671

[18] Poirel L, Cattoir V, Soares A, Soussy CJ, Nordmann P. 2007. Novel ambler class a beta-lactamase LAP-1 and its association with the plasmid-mediated quinolone resistance determinant QnrS1. Antimicrob Agents Chemother 51:631–637. doi:10.1128/AAC.01082-06 17116662 PMC1797722

[19] McClelland M, Sanderson KE, Spieth J, Clifton SW, Latreille P, Courtney L, Porwollik S, Ali J, Dante M, Du F, et al.. 2001. Complete genome sequence of Salmonella enterica serovar Typhimurium LT2. Nat New Biol 413:852–856. doi:10.1038/35101614 11677609

